# FBP1 orchestrates keratinocyte proliferation/differentiation and suppresses psoriasis through metabolic control of histone acetylation

**DOI:** 10.1038/s41419-024-06706-6

**Published:** 2024-06-04

**Authors:** Pengfei Zhang, Ju Yang, Xiong Liu, Congshu Huang, Yuandong Tao, Pan Shen, Zhijie Bai, Chengrong Xiao, Lei Zhou, Gaofu Li, Li Zhang, Wei Zhou, Yue Gao

**Affiliations:** 1 Department of Pharmaceutical Sciences, Beijing Institute of Radiation Medicine, Beijing, 100850 China; 2 Department of Dermatology, The General Hospital of Western Theater Command PLA, Chengdu, Sichuan 610083 China; 3 Department of Information, The PLA Center for Disease Control and Prevention, Beijing, China; 4 Department of Pediatric Urology, The Seventh Medical Center of Chinese PLA General Hospital, Beijing, China

**Keywords:** Cell biology, Diseases

## Abstract

Keratinocyte proliferation and differentiation in epidermis are well-controlled and essential for reacting to stimuli such as ultraviolet light. Imbalance between proliferation and differentiation is a characteristic feature of major human skin diseases such as psoriasis and squamous cell carcinoma. However, the effect of keratinocyte metabolism on proliferation and differentiation remains largely elusive. We show here that the gluconeogenic enzyme fructose-1,6-bisphosphatase 1 (FBP1) promotes differentiation while inhibits proliferation of keratinocyte and suppresses psoriasis development. *FBP1* is identified among the most upregulated genes induced by UVB using transcriptome sequencing and is elevated especially in upper epidermis. *Fbp1* heterozygous mice exhibit aberrant epidermis phenotypes with local hyperplasia and dedifferentiation. Loss of *FBP1* promotes proliferation and inhibits differentiation of keratinocytes in vitro. Mechanistically, *FBP1* loss facilitates glycolysis-mediated acetyl-CoA production, which increases histone H3 acetylation at lysine 9, resulting in enhanced transcription of proliferation genes. We further find that the expression of *FBP1* is dramatically reduced in human psoriatic lesions and in skin of mouse imiquimod psoriasis model. *Fbp1* deficiency in mice facilitates psoriasis-like skin lesions development through glycolysis and acetyl-CoA production. Collectively, our findings reveal a previously unrecognized role of FBP1 in epidermal homeostasis and provide evidence for FBP1 as a metabolic psoriasis suppressor.

## Introduction

The epidermis of mammalian skin serves as a water-impermeable barrier which is essential for keeping harmful insults out. Cells from a basal proliferative layer of epidermis commit to differentiate and move upward during normal tissue homeostasis or after exposure to stimuli such as ultraviolet light. Disturbances in epidermal homeostasis are associated with various skin diseases such as psoriasis. Psoriatic lesions are characterized by epidermal acanthosis (thickening of viable layers), hyperkeratosis (thickened cornified layer), and parakeratosis (cell nuclei present in the cornified layer), which are due to increased proliferation and aberrant terminal differentiation of keratinocytes [1, 2]. The keratinocyte response is triggered by dysregulated cellular immune system, with T cells, dendritic cells, and various immune-related cytokines and chemokines implicated in pathogenesis [2].

Glycolysis, as an important metabolic pathway in cells, provides energy, reducing equivalents and materials for the synthesis of cellular biomass to maintain cell division. It has been proven that epidermis is a glycolytic tissue [3, 4]. Recent evidences demonstrate that the glycolytic enzyme 6-phosphofructo-2-kinase/fructose-2,6-biphosphatase 3 (PFKFB3) is required for proliferation and inhibits differentiation in epidermal keratinocytes, implicating glycolysis directly regulates keratinocyte proliferation and differentiation [5]. The glycolytic enzymes are also increasingly proven to participate in the pathogenesis of psoriasis, as psoriatic lesions exhibit increased proliferating keratinocytes [6]. For example, glucose transporter 1 (GLUT1) is elevated in psoriatic epidermis from the patient and imiquimod (IMQ) induced psoriasis mouse model, genetic and pharmacological *Glut1* inactivation decreased hyperplasia in mouse models of psoriasis-like disease [7]; pyruvate kinase M2 contributes to psoriasis progression by promoting proliferation and mediating interleukin-17 signaling in keratinocytes [8, 9]. These studies have examined aerobic glycolysis in keratinocyte proliferation and differentiation; however, gluconeogenesis has not been fully studied.

As a rate-limiting gluconeogenic enzyme, fructose-1,6-bisphosphatase (FBP1), catalyzes the hydrolysis of fructose-1,6-bisphosphate to fructose 6-phosphate. Recent studies demonstrate that FBP1 also functions as a protein phosphatase to dephosphorylate histone H3 and IκBα [10, 11]. An autosomal recessive inherited disorder of *FBP1* deficiency is characterized by hypoglycemia and lactic acidosis, which often results in sudden infant death [12]. FBP1 is increasingly identified as a tumor suppressor as loss of *FBP1* expression has been shown to promote tumor progression by enhancing aerobic glycolysis, thereby resulting in poor prognosis in patients with clear cell renal cell carcinoma, breast cancer and hepatocellular carcinoma [13–16]. FBP1 also plays important role in pathophysiology of diabetes, obesity and insulin hyperresponsiveness [17–20]. However, to our knowledge, the role of FBP1 in epidermal homeostasis and psoriasis has not been studied.

Here we identify *FBP1* is among the most upregulated genes induced by UVB and is elevated especially in upper epidermis. Functional studies demonstrate *Fbp1* loss disturbs epidermal homeostasis with increased hyperplasia and reduced differentiation using genetic mice model. Mechanistically, *FBP1* depletion promotes glycolysis-mediated acetyl-CoA production, which increases histone acetylation and subsequent growth-related genes transcription in keratinocytes. We also find *FBP1* is dramatically reduced in human psoriatic lesions and *Fbp1* deficient mice are more prone to develop IMQ-induced psoriasis-like skin lesions, demonstrating FBP1 participates in psoriasis pathogenesis. Our results uncover a previously unrecognized role of FBP1 in epidermal homeostasis and suggest that restoring FBP1 in psoriatic lesions might be a promising approach for treating psoriasis.

## Result

### UVB irradiation causes epidermal differentiation and metabolic changes in human epidermal equivalents

**Fig. 1:**
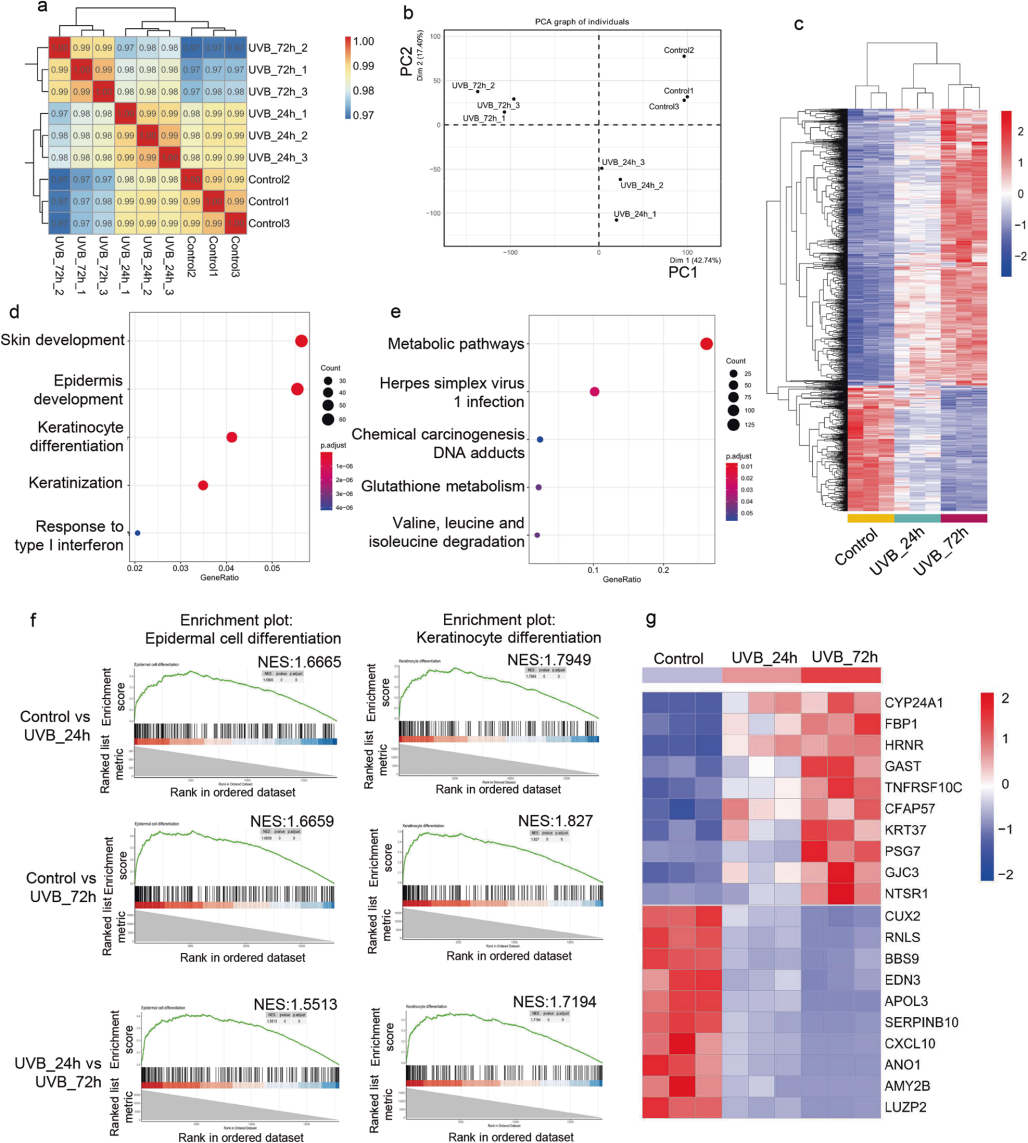
UVB irradiation causes epidermal differentiation and metabolic changes in human epidermal equivalents. **a** Sample correlation analysis of the transcriptome of human epidermal equivalents from the control group, UVB_24 h group and UVB_72 h group. **b** Principal component analysis of the transcriptome of human epidermal equivalents from the control group, UVB_24 h group and UVB_72 h group. **c** Clustering heatmap of the 1227 differentially expressed genes based on FPKM. **d**, **e** Over-representation analysis of GO (**d**) and KEGG pathways (**e**) based on the 1227 differentially expressed genes. The size indicates the gene numbers and the color corresponds to the *P* value. **f** Gene set enrichment analysis (GSEA) of differentially expressed genes in the UVB_24 h group relative to the control group, UVB_72 h group relative to the control group and UVB_72 h group relative to the UVB_24 h group. **g** Expression heatmap of the top 10 changed genes.

To investigate the transcriptional response to UVB radiation in human epidermis, we utilized human primary keratinocytes to generate human epidermal equivalents, which were then irradiated with 500 mJ/cm^2^ UVB and collected 24 h (UVB_24 h) or 72 h (UVB_72 h) after irradiation. The non-irradiated epidermis serves as control. Gene expression in irradiated and non-irradiated epidermis was characterized by RNA-Seq technique. A total of 17,929 genes were detected in these samples. Samples of the same group clustered closely evaluated by Spearman’s correlation coefficient method, suggesting high fidelity of RNA-Seq data (Fig. 1a). Principal component analysis (PCA) demonstrated a clear separation of transcriptional signatures induced by UVB irradiation (Fig. 1b). To further characterize differentially expressed genes from RNA-Seq data, we set the threshold with Q-value < 0.001 and |log2FC| > 1 by DESeq2 R package. A total of 2672 genes were identified differentially expressed among three groups. We further narrowed down the differentially expressed genes to 1515 by excluding genes of average Fragment Per Kilobase of transcript, per Million mapped reads (FPKM) <1. Among the 1515 genes, 25.3% (383/1515) exhibited a continuously increased expression pattern while 55.7% (844/1515) exhibited a continuously decreased expression pattern (Fig. 1c). Over-representation analysis of Gene Ontology (GO) and Kyoto Encyclopedia of Genes and Genomes (KEGG) pathways using the 1227 continuously changing genes revealed a significant enrichment in the skin development, epidermis development, keratinocyte differentiation (Fig. 1d) and metabolic pathways (Fig. 1e). Gene set enrichment analysis based on the entire transcriptome data identified GO enrichment in epidermal cell differentiation and keratinocyte differentiation in all three comparisons (Fig. 1f). Thus, UVB irradiation induces keratinocyte differentiation and metabolic changes in human epidermal equivalents.

### FBP1 expression is induced by UVB irradiation

**Fig. 2:**
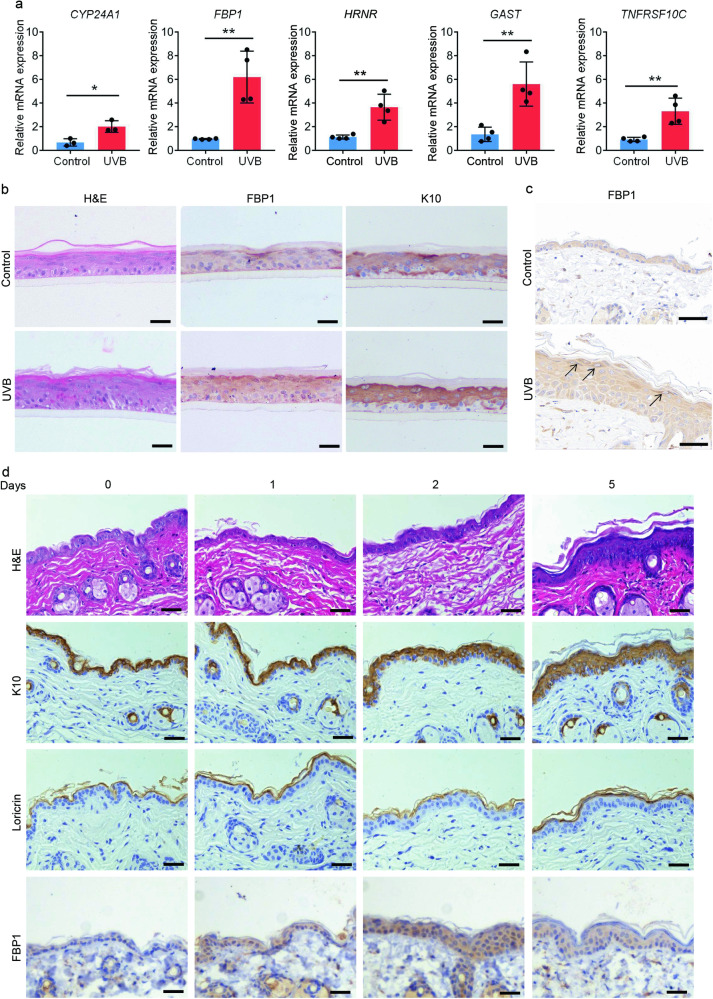
*FBP1* expression is induced by UVB irradiation. **a** Relative mRNA levels of *CYP24A1*, *FBP1*, *HRNR*, *GAST* and *TNFRSF10C* in control (0 mJ/cm^2^) and UVB-irradiated (500 mJ/cm^2^) human epidermal equivalents collected 72 h after irradiation, *n* = 3 or 4 independent experiments. **b** Representative H&E staining or immunohistochemical staining (FBP1, K10) of control (0 mJ/cm^2^) or UVB (500 mJ/cm^2^) irradiated human epidermal equivalents collected 72 h after irradiation. Scale bars: 50 μm. **c** Representative immunohistochemical staining for FBP1 of control (0 mJ/cm^2^) or UVB (500 mJ/cm^2^) irradiated mice skin collected 72 h after irradiation. Scale bars: 50 μm. Arrows indicated deep-stained upper epidermis. **d** Representative H&E staining or immunohistochemical staining of mice skin collected at indicated days after irradiation. Scale bars: 50 μm. Data are shown as mean ± s.d. Statistical analyses in (**a**) were performed with Student’s *t*-tests. *p < 0.05, **p < 0.01.

We further examined the top 10 upregulated genes induced by UVB irradiation (Fig. 1g). Among these genes, *CYP24A1* (*cytochrome P450 family 24 subfamily A member 1*) is a vitamin D3 hydroxylating enzyme which can be induced by UVB irradiation in wavelength-dependent manner in human keratinocytes [21–23]. *Hornerin* (*HRNR)* is a component of the epidermal cornified cell envelopes and its expression is induced by UVB irradiation [24, 25]. Thus, the increased expression of UVB-responsive genes detected in irradiated samples indicated our transcriptomic data were convincing. Indeed, the RT-qPCR analysis confirmed the increased transcription levels of *CYP24A1, FBP1, HRNR, GAST* (*gastrin*) and *TNFRSF10C* (*TNF receptor superfamily member 10c*) (Fig. 2a). As FBP1 is a rate-limiting gluconeogenic enzyme and its role in epidermis was unknown, we selected FBP1 for further studies. UVB irradiation promoted the stratification process, increased the thickness, and induced *FBP1* expression in human epidermis equivalents (Fig. 2b). *Fbp1* expression was also upregulated in UVB-irradiated mice skin, especially in upper epidermis (Fig. 2c). We further detected FBP1 levels in mice skin at various times after UVB irradiation. UVB promoted epidermal differentiation and inflammation as the expression of the differentiation marker *keratin10* (*K10*), *loricrin cornified envelope precursor protein* (*Loricrin*) and the cytokines *interleukin 1 beta* (*Il1b*), *interleukin 6* (*Il6*), *interleukin 8* (*Il8*) were elevated (Fig. 2d and Supplementary Fig. 1a, b). Meanwhile, FBP1 level was slightly elevated 1 day after UVB irradiation and significantly elevated 2 days after UVB irradiation (Fig. 2d and Supplementary Fig. 1b). In summary, UVB induced *Fbp1* expression, concomitant with the increased epidermal differentiation and inflammation.

We also investigated the regulation of *FBP1* expression. As IL-6 is induced by UVB irradiation and is known to activate the expression of *pyruvate carboxylase 1*, another gluconeogenesis enzyme, in the liver [26], we hypothesized IL-6 might be one of the regulators of FBP1. However, FBP1 levels were not changed upon *IL-6* knockdown (Supplementary Fig. 1c). Additionally, the transcription factor for PFKFB3, p63 [5], also did not control *FBP1* expression (Supplementary Fig. 1c). As UVB activates p38 mitogen-activated protein kinase (MAPK) pathways and NF-κB pathways [27, 28], we further detected whether theses pathways participated in regulating FBP1. Indeed, inhibition of p38 MAPK pathways by adezmapimod or inhibition of NF-κB pathways by BAY 11-7082 slightly downregulated *FBP1* expression, demonstrating these two pathways partially regulate FBP1 levels (Supplementary Fig. 1d).

### FBP1 is essential for epidermal homeostasis and response to UVB irradiation

**Fig. 3:**
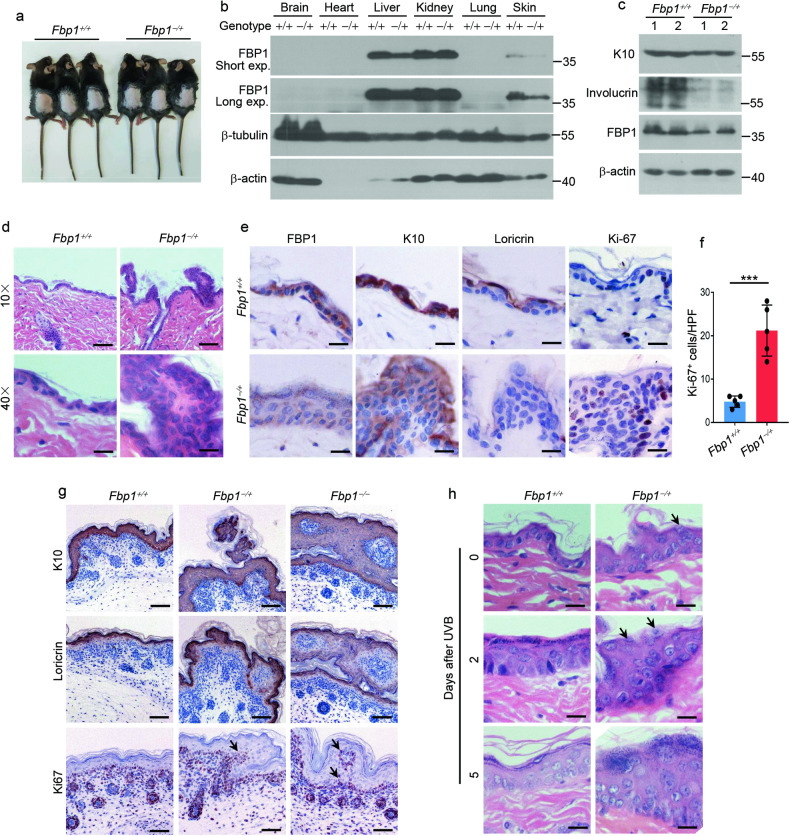
FBP1 is essential for epidermal homeostasis and response to UVB irradiation. **a** Photos of 6-week-old *Fbp1*^*+*/*+*^ and *Fbp1*^*−*/*+*^ mice. **b** Western blot analysis of the expression of FBP1, β-tubulin and β-actin in indicated organs obtained from *Fbp1*^*+*/*+*^ and *Fbp1*^−/*+*^ mice. Exp exposure. **c** Western blot analysis of the expression of K10, involucrin, FBP1 and β-actin in skin tissue from *Fbp1*^*+*/*+*^ and *Fbp1*^−/*+*^ mice. **d** Representative H&E staining of skin from 6-week-old *Fbp1*^*+*/*+*^ and *Fbp1*^*−*/*+*^ mice. Scale bars in upper images: 100 μm, scale bars in lower images: 25 μm. **e** Immunohistochemical staining for FBP1, K10, loricrin and Ki-67 of skin from 6-week-old *Fbp1*^*+*/*+*^ and *Fbp1*^*−*/*+*^ mice. Scale bars: 25 μm. **f** The quantification of Ki-67^+^ keratinocytes in skin sections from 6-week-old *Fbp1*^*+*/*+*^ and *Fbp1*^−/*+*^ mice, *n* = 3 mice per genotype. HPF high-power field. **g** Immunohistochemical staining for K10, loricrin and Ki-67 of skin from neonatal *Fbp1*^*+*/*+*^, *Fbp1*^−/*+*^ and *Fbp1*^−/−^ mice. Scale bars: 100 μm. Arrows indicated protruding Ki-67^+^ basal keratinocytes. **h** Representative H&E staining of skin from 6-week-old *Fbp1*^+/+^ and *Fbp1*^−/+^ mice collected at indicated days after irradiation. Scale bars: 50 μm. Arrows indicated retained cell nuclei in the cornified layer. Data are shown as mean ± s.d. Statistical analyses in (**f**) were performed with Student’s *t*-tests. ***p < 0.001.

To elucidate the role of FBP1 in epidermis, we utilized genetic mice model. By using CRISPR-Cas9 technology, we deleted the exon 2–4 (5120 bp) of the murine *Fbp1* gene (Supplementary Fig. 2a). We designed primers outside (F1, R1) and inside (F2, R2) the deletion region (Supplementary Fig. 2b,c), and verified *Fbp1* genotypes by PCR (Supplementary Fig. 2d). Although *Fbp1* homozygous (*Fbp1*^*−*/−^) newborn mice were morphologically indistinguishable from WT littermates (*Fbp1*^*+*/*+*^) (Supplementary Fig. 2e), they died several days after birth, which might be caused by hypoglycemia and lactic acidosis observed in *FBP1* deficient human infants [12]. Thus, we further analyzed the *Fbp1* heterozygous mice (*Fbp1*^*−*/*+*^). Six-week-old heterozygous mice exhibited normal morphology compared with their WT littermates (Fig. 3a). Western blot demonstrated that *Fbp1* was strongly expressed in liver and kidney, weakly expressed in skin and not expressed in brain, heart and lung (Fig. 3b). *Fbp1* expression was ~50% lower in heterozygous liver and skin (Fig. 3b). Surprisingly, skin of *Fbp1* heterozygous mice exhibited focal hyperplasia with disorganized keratinocytes and enlarged nuclei (Fig. 3d). The expression of epidermal *K10* and *Loricrin* were decreased while proliferation marker *Ki-67* was increased in these skin lesions, suggesting decreased epidermal differentiation and increased proliferation (Fig. 3c, e, f). We further analyzed skin of neonatal *Fbp1* homozygous mice and also found disorganized focal lesions (Fig. 3g), demonstrating that FBP1 is essential for maintaining skin homeostasis. We next tested whether FBP1 was necessary for the epidermal response to UVB irradiation. Many areas of *Fbp1* heterozygous mice skin showed abnormal stratification with retained cell nuclei in the cornified layer after UVB irradiation (Fig. 3h). MB05032, a specific inhibitor of FBP1 [29], also reduced the expression of keratin10 and loricrin in human epidermal equivalents with or without UVB treatment (Supplementary Fig. 2f, g). In summary, FBP1 is essential for epidermal homeostasis and response to UVB irradiation.

### FBP1 promotes differentiation and inhibits proliferation of keratinocytes

**Fig. 4:**
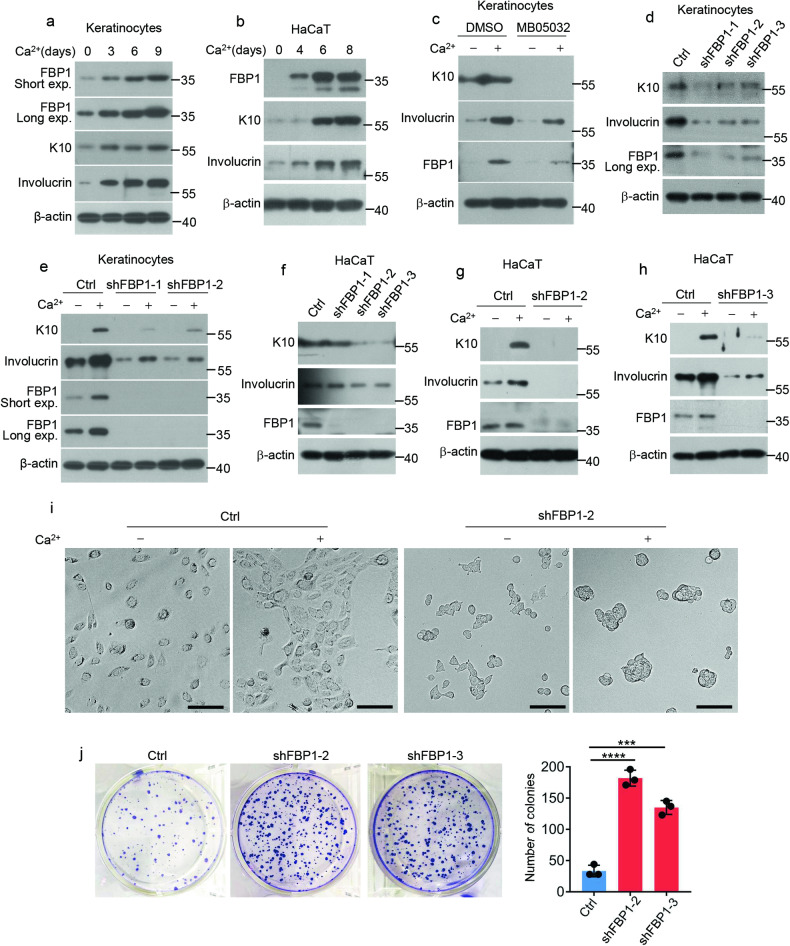
FBP1 promotes differentiation and inhibits proliferation of keratinocytes. **a** Human primary keratinocytes were treated with CaCl_2_ (1.2 mM) for 0, 3, 6 or 9 days before collection. The indicated proteins were detected by western blots. Exp, exposure. **b** HaCaT cells were treated with CaCl_2_ (1.2 mM) for 0, 4, 6 or 8 days before collection. The indicated proteins were detected by western blots. **c** Human primary keratinocytes were treated with DMSO or 40 μM MB05032 for 2 days and then exposed to 0.06 mM (−) or 1.2 mM (+) calcium for 3 days before collection. The indicated proteins were detected by western blots. **d** Ctrl shRNA or *FBP1* shRNAs (shFBP1-1, shFBP1-2, shFBP1-3) were packaged into lentiviral particles and transduced into keratinocytes. The positive clones were selected by puromycin (2 µg/ml). The indicated proteins were detected by western blots. Exp, exposure. **e** Keratinocytes stably expressing Ctrl shRNA or *FBP1* shRNAs (shFBP1-1, shFBP1-2) were exposed to 0.06 mM (−) or 1.2 mM (+) calcium for 3 days before collection. The indicated proteins were detected by western blots. Exp, exposure. **f** Ctrl shRNA or *FBP1* shRNAs (shFBP1-1, shFBP1-2, shFBP1-3) were packaged into lentiviral particles and transduced into HaCaT cells. The positive clones were selected by puromycin (2 µg/ml). The indicated proteins were detected by western blots. **g**, **h** HaCaT cells stably expressing Ctrl, shFBP1-2 (**g**) or shFBP1-3 (**h**) were exposed to 0.06 mM (−) or 1.2 mM (+) calcium for 6 days before collection. The indicated proteins were detected by western blots. **i** HaCaT cells stably expressing Ctrl or shFBP1-2 were exposed to 0.06 mM (−) or 1.2 mM (+) calcium for 3 days before observation by microscopy. Scale bars: 50 µm. **j** HaCaT cells stably expressing Ctrl or shFBP1-2 were plated (1000 cells/well) and cultured for 2 weeks. The colonies were stained with 0.1% crystal violet and counted, *n* = 3 independent experiments. Data are shown as mean ± s.d. Statistical analyses in (**j**) were performed with Student’s *t*-tests. ***p < 0.001, ****p < 0.0001.

As *Fbp1* deficient mice exhibits disturbed epidermal homeostasis with increased hyperplasia and reduced differentiation, we hypothesized that FBP1 might participate in keratinocytes differentiation and proliferation. We next utilized human primary keratinocytes and HaCaT cells to clarify the role of FBP1. FBP1 gradually accumulated during calcium-induced differentiation of human primary keratinocytes and HaCaT cells (Fig. 4a, b), implicating FBP1 was involved in keratinocytes differentiation. To investigate whether FBP1 regulated keratinocytes differentiation, we treated human primary keratinocytes with DMSO or MB05032 and induced differentiation with calcium. Compared with DMSO, MB05032 significantly suppressed the expression of differentiation genes (Fig. 4c). We further depleted intracellular *FBP1* using short hairpin RNAs (shFBP1) (Fig. 4d). *FBP1* depletion significantly inhibited K10 and involucrin levels under basal condition and calcium-induced differentiation condition (Fig. 4d, e). We also depleted *FBP1* in immortalized HaCaT keratinocytes (Fig. 4f). Similarly, both K10 and involucrin levels were significantly inhibited in shFBP1 cells compared with control cells cultured in basal medium or differentiation medium (Fig. 4f–h). Interestingly, we found morphology of HaCaT cells changed dramatically upon *FBP1* depletion. Control cells exhibited polygonal shape and changed from scattered to closely arranged state with calcium treatment, while shFBP1 cells exhibited a small, rounded shape and formed clusters upon calcium treatment (Fig. 4i), indicating the dedifferentiation of shFBP1 cells. We further tested whether shFBP1 cells showed proliferation changes. The growth of shFBP1 HaCaT cells markedly increased compared with that of control cells, on the basis of crystal violet staining and cell numbers (Fig. 4j), indicating FBP1 inhibited keratinocytes proliferation. Additionally, the expression of cytokines *IL1B* and *IL-6* was suppressed in shFBP1 cells under basal condition or after UVB irradiation (Supplementary Fig. 3a–c). Collectively, these results demonstrate that FBP1 promotes differentiation while inhibits proliferation of keratinocytes.

### FBP1 regulates keratinocyte proliferation/differentiation in glycolysis-dependent manner

**Fig. 5:**
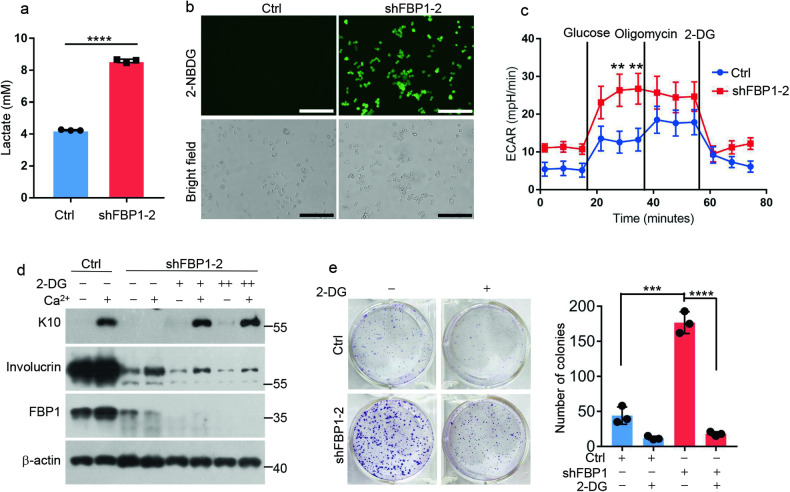
FBP1 regulates keratinocyte proliferation/differentiation in glycolysis-dependent manner. **a** Ctrl or shFBP1-2 HaCaT cells were incubated for an additional 24 h in growth medium 3 days after plating. Medium was collected and lactate content was measured, *n* = 3 independent experiments. **b** Ctrl or shFBP1-2 HaCaT cells were treated with 2-NBDG (100 μM) for 30 min and images were taken with an excitation wavelength of 485 nm and an emission wavelength of 535 nm. Scale bars: 100 µm. **c** Extracellular acidification rate (ECAR) of Ctrl and shFBP1-2 HaCaT cells were detected using a Seahorse XF extracellular flux instrument, *n* = 3 independent experiments. **d** Ctrl or shFBP1-2 HaCaT cells were treated with 0 mM (−), 1 mM (+) or 2 mM (++) 2-DG for 2 days, and then exposed to 0.06 mM (−) or 1.2 mM (+) calcium for 6 days before collection. The indicated proteins were detected by western blots. **e** Ctrl or shFBP1-2 HaCaT cells were plated (1000 cells/well) and cultured with or without 1 mM 2-DG for 2 weeks. The colonies were stained with 0.1% crystal violet and counted, *n* = 3 independent experiments. Data are shown as mean ± s.d. Statistical analyses in (**a**), (**c**) and (**e**) were performed with Student’s *t*-tests. **p < 0.01, ***p < 0.001, ****p < 0.0001.

As a rate-limiting gluconeogenic enzyme, FBP1 inhibits glucose uptake and glycolysis. To determine whether FBP1 regulates glycolytic metabolism in keratinocytes, we compared glycolysis between control and shFBP1 HaCaT cells. Depletion of *FBP1* dramatically promoted lactate production and glucose uptake (Fig. 5a, b). To further examine the ability of shFBP1 cells to metabolize glucose, we measured the extracellular acidification rate (ECAR) using a Seahorse XF extracellular flux instrument. ShFBP1 cells exhibited enhanced ECAR compared with control cells upon the addition of glucose to the culture media (Fig. 5c). Furthermore, injection of oligomycinA, which inhibits mitochondrial ATP production and induces maximal glycolytic rates, failed to further increase ECAR in shFBP1 cells, indicating shFBP1 cells maintained the maximum glycolytic capacity in the presence of glucose (Fig. 5c). These experiments suggested that *FBP1* loss enhanced glycolysis in keratinocytes. To investigate whether FBP1 regulated keratinocytes proliferation/differentiation in glycolysis-dependent manner, we treated shFBP1 cells with 2-Deoxy-D-glucose (2-DG), a glucose analog that acts as a competitive inhibitor of glucose metabolism. Inhibition of *K10* expression in shFBP1 cells was fully reversed by administration of 2-DG (Fig. 5d). However, the involucrin levels were not altered by 2-DG treatment in shFBP1 cells. One possible reason was that 2-DG induced the unfolded protein response and affected protein translation as 2-DG is also chemically identical with 2-deoxymannose [5, 30]. Meanwhile, the increased growth of shFBP1 HaCaT cells was suppressed by 2-DG treatment, assessed by crystal violet staining and cell numbers (Fig. 5e). Thus, FBP1 regulates keratinocytes proliferation/differentiation through glycolysis.

### *FBP1* loss promotes acetyl-CoA production and histone acetylation

**Fig. 6:**
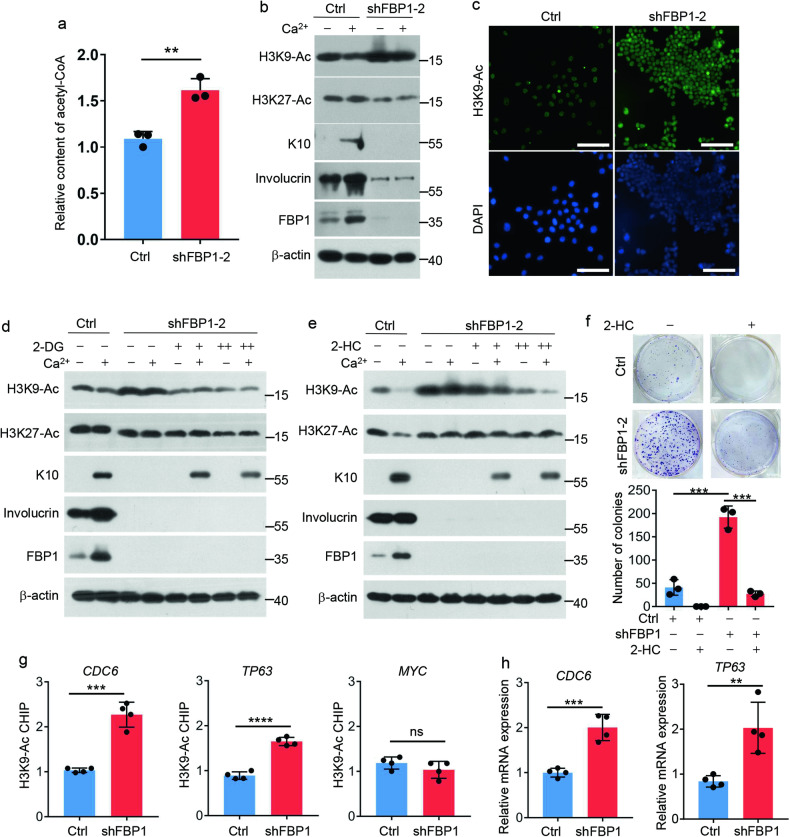
*FBP1* loss promotes acetyl-CoA production and histone acetylation. **a** 1 × 10^6^ Ctrl or shFBP1-2 HaCaT cells were harvested and acetyl-CoA levels in HaCaT cells were detected by acetyl-CoA Assay. Values were expressed as fold differences compared to Ctrl, *n* = 3 independent experiments. **b** Ctrl or shFBP1-2 HaCaT cells were exposed to 0.06 mM (−) or 1.2 mM (+) calcium for 6 days before collection. The indicated proteins were detected by western blots. **c** Immunostaining for H3K9-Ac (green) in Ctrl or shFBP1-2 HaCaT cells. The nuclei were stained by DAPI. Scale bars: 100 μm. **d** Ctrl or shFBP1-2 HaCaT cells were treated with 0 mM (−), 1 mM (+) or 2 mM (++) 2-DG for 2 days, and then exposed to 0.06 mM (−) or 1.2 mM (+) calcium for 6 days before collection. The indicated proteins were detected by western blots. **e** Ctrl or shFBP1-2 HaCaT cells were treated with 0 mM (−), 2.5 mM (+) or 5 mM (++) 2-HC for 2 days, and then exposed to 0.06 mM (−) or 1.2 mM (+) calcium for 6 days before collection. The indicated proteins were detected by western blots. **f** Ctrl or shFBP1-2 HaCaT cells were plated (1000 cells/well) and cultured with or without 2.5 mM 2-HC for 2 weeks. The colonies were stained with 0.1% crystal violet and counted, *n* = 3 independent experiments. **g** H3K9-ac abundance on indicated genes in Ctrl or shFBP1-2 HaCaT cells was detected by H3K9-ac immunoprecipitation and qPCR. Values were expressed as fold differences compared to Ctrl, *n* = 4 independent experiments. **h** Relative mRNA levels of *CDC6* and *TP63* in Ctrl and shFBP1 HaCaT cells, *n* = 4 independent experiments. Data are shown as mean ± s.d. Statistical analyses in (**a**), (**f**), (**g**) and (**h**) were performed with Student’s *t*-tests. **p < 0.01, ***p < 0.001, ****p < 0.0001, ns not significant.

In mammalian cells, glycolysis drives synthesis of citrate, which is converted to acetyl-CoA by ATP-citrate lyase (ACLY). As a critical second messenger, acetyl-CoA controls key cellular processes, including energy metabolism, mitosis, and autophagy through influencing the acetylation profile of several proteins [31]. Thus, we detected whether enhanced glycolysis promoted acetyl-CoA production in *FBP1*-depleted cells. Indeed, acetyl-CoA levels were higher in shFBP1 cells compared to control cells (Fig. 6a). Acetyl-CoA was shown to be rate limiting for histone acetylation [31], thus, we speculated increased acetyl-CoA in shFBP1 cells might affect the histone acetylation state. To test this hypothesis, we detected global histone 3 acetylation at lysine K9 (H3K9-Ac) and K27 (H3K27-Ac), marks that are associated with chromatin opening and transcription. We observed increased H3K9-Ac in shFBP1 cells compared with control cells (Fig. 6b, c). Indeed, intracellular acetyl-CoA levels were reported to induce the Gcn5-catalyzed H3K9-Ac [32, 33]. However, H3K27-Ac had no obvious changes upon *FBP1* depletion, even slightly downregulated (Fig. 6b). These results demonstrated that *FBP1* loss promoted acetyl-CoA production and H3K9-Ac. Next, we sought to link the change in histone acetylation to the shift in glycolysis and acetyl-CoA in *FBP1*-depleted cells. Inhibition of glycolysis by 2-DG or inhibition of acetyl-CoA production by 2-hydroxycitrate (2-HC), a competitive inhibitor of ACLY, decreased H3K9-Ac levels in shFBP1 cells (Fig. 6d,e), suggesting increased histone acetylation in *FBP1*-depleted cells was through glycolysis-mediated acetyl-CoA. Furthermore, 2-HC significantly reversed both the suppressed differentiation and accelerated proliferation in shFBP1 cells, as detected by *K10* expression and clone numbers (Fig. 6e, f), demonstrating FBP1 regulates keratinocytes proliferation/differentiation in acetyl-CoA-dependent manner.

Intracellular acetyl-CoA levels had been reported to induce the H3K9-Ac at genes important for growth, thereby enabling their rapid transcription and commitment to growth [32]. To examine the abundance of H3K9-Ac at growth genes, we performed chromatin immunoprecipitation–qPCR (ChIP–qPCR) analysis. Compared with control cells, H3K9-Ac at *CDC6* (*cell division cycle 6*) and *TP63* (*tumor protein p63*) genes were more abundant in shFBP1 cells (Fig. 6g), in accord with the increased transcript levels of *CDC6* and *TP63* in shFBP1 cells (Fig. 6h), which explained the rapid proliferation and dedifferentiation phenotype of *FBP1*-depleted keratinocytes. Together, these results demonstrate that *FBP1* loss promotes acetyl-CoA production, which increases H3K9-Ac at growth-related genes such as *CDC6* and *TP63*, thus enabling their rapid transcription and cell proliferation.

### *Fbp1* deficiency aggravates psoriasis models

**Fig. 7:**
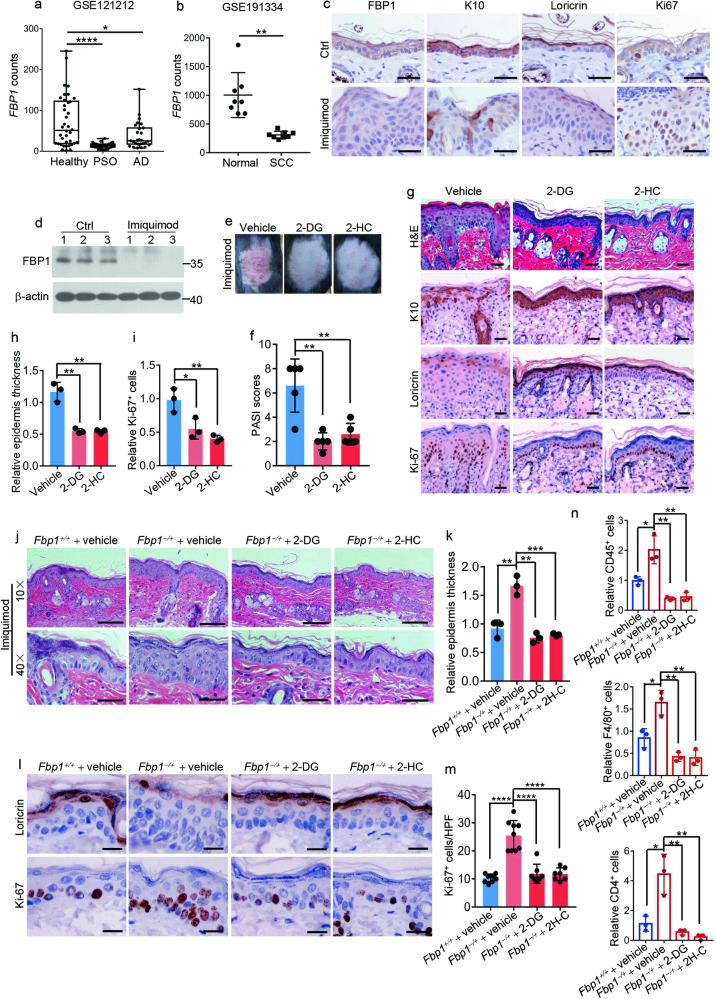
*Fbp1* deficiency aggravates psoriasis models. **a** *FBP1* levels in healthy skin, psoriatic lesions or atopic dermatitis lesions based on RNA-Seq datasets from public databases GSE121212. **b** *FBP1* levels in normal skin or squamous cell carcinoma based on GSE191334. **c** Immunohistochemical staining for FBP1, K10, loricrin and Ki-67 of skin from Ctrl or imiquimod-treated mice. Scale bars: 50 μm. **d** Western blot analysis of the expression of FBP1 and β-actin in skin tissues from Ctrl or imiquimod-treated mice. **e** Photos of skin lesions from vehicle, 2-DG or 2-HC pretreated mice after 8 days treatment with imiquimod. **f** The Psoriasis Area and Severity Index (PASI) score for imiquimod-induced skin lesions from vehicle, 2-DG or 2-HC pretreated mice, *n* = 5 mice per group. **g** Representative H&E staining or immunohistochemical staining for K10, loricrin and Ki-67 of imiquimod-induced skin lesions from vehicle, 2-DG or 2-HC pretreated mice. Scale bars: 50 μm. **h** Relative epidermis thickness for imiquimod-induced skin lesions from vehicle, 2-DG or 2-HC pretreated mice, *n* = 3 mice per group. **i** Relative Ki-67^+^ keratinocytes for imiquimod-induced skin lesions from vehicle, 2-DG or 2-HC pretreated mice, *n* = 3 mice per group. **j** Representative H&E staining of skin lesions from vehicle pretreated *Fbp1*^+/+^ mice, vehicle, 2-DG or 2-HC pretreated *Fbp1*^−/+^ mice after 5 days treatment with imiquimod. Scale bars in upper images: 200 μm, scale bars in lower images: 50 μm. **k** Relative epidermis thickness for imiquimod-induced skin lesions from vehicle pretreated *Fbp1*^+/+^ mice, vehicle, 2-DG or 2-HC pretreated *Fbp1*^−/+^ mice, *n* = 3 mice per group. **l** Immunohistochemical staining for loricrin and Ki-67 of imiquimod-induced skin lesions from vehicle pretreated *Fbp1*^+/+^ mice, vehicle, 2-DG or 2-HC pretreated *Fbp1*^−/+^ mice. Scale bars: 25 μm. **m** The quantification of Ki-67^+^ keratinocytes in imiquimod-induced skin lesions from vehicle pretreated *Fbp1*^+/+^ mice, vehicle, 2-DG or 2-HC pretreated *Fbp1*^−/+^ mice, *n* = 3 mice per group. HPF high-power field. **n** Relative leukocytes (CD45^+^), macrophages (F4/80^+^) or T cells (CD4^+^) numbers of imiquimod-induced skin lesions (8 days) from vehicle pretreated *Fbp1*^+/+^ mice, vehicle, 2-DG or 2-HC pretreated *Fbp1*^−/+^ mice, *n* = 3 mice per group. Data are shown as mean ± s.d. Statistical analyses in (**a**), (**b**), (**f**), (**h**), (**i**), (**k**), (**m**), (**n**) were performed with Student’s *t*-tests. *p < 0.05, **p < 0.01, ***p < 0.001, ****p < 0.0001.

Psoriasis is a chronic cytokine-driven, inflammatory skin disease characterized by hyperplasia and abnormal differentiation of the epidermis that presents as thickened and scaly plaques. Keratinocyte hyperproliferation is triggered pathologically in psoriasis. To explore whether FBP1 participated in psoriasis pathogenesis, we examined *FBP1* expression in biopsies from people with psoriasis and healthy controls using a publicly available RNA-seq dataset (Gene Expression Omnibus database: accession no. GSE121212). In comparison with healthy controls, *FBP1* expression significantly decreased in skin lesions from patients with psoriasis, and to a less extent, decreased in skin lesions from patients with atopic dermatitis (Fig. 7a). Meanwhile, the *LORICRIN* expression was downregulated while the *CDC6* expression was upregulated in skin lesions from patients with psoriasis, demonstrating reduced differentiation and increased proliferation in psoriatic lesions (Supplementary Fig. 4a, b). Additionally, we also found *FBP1* and *CDC6* levels were dramatically reduced in skin lesions from patients with squamous cell carcinoma (SCC) using dataset GSE191334 (Fig. 7b and Supplementary Fig. 4c), implicating FBP1 might also participate in SCC development. We further established the mice model of psoriasis through topical application of imiquimod. The imiquimod-treated mice developed psoriasiform lesions characterized by epidermal acanthosis (Supplementary Fig. 4d), with reduced expression of differentiation marker *K10*, *Loricrin* and increased expression of proliferation marker *Ki-67* (Fig. 7c). *Fbp1* expression was downregulated in psoriasiform lesions of imiquimod-treated mice (Fig. 7c, d). The imiquimod-induced psoriasiform lesions were alleviated by topical application of 2-DG or 2-HC (Fig. 7e, f) compared to vehicle-treated mice, demonstrating glycolysis and acetyl-CoA production participated in this progress. 2-DG or 2-HC application also decreased psoriasiform hyperplasia, epidermis thickness and proliferating (Ki-67^+^) cells, while increased differentiation markers (*K10*, *Loricrin*) expression in psoriasiform lesions (Fig. 7g–i) compared to vehicle-treated mice. Furthermore, the number of infiltrated leukocytes (CD45^+^), macrophages (F4/80^+^) and T cells (CD4^+^) in psoriasiform skin lesions were also significantly reduced by 2-DG or 2-HC treatment (Supplementary Fig. 4e, f) compared to vehicle-treated mice. Thus, inhibition of glycolysis or acetyl-CoA production largely normalized keratinocyte proliferation/differentiation and decreased immune cell infiltration in mice psoriasis model.

We further tested whether *Fbp1* deficiency affect the development of pathological hyperplasia in psoriasis models. *Fbp1*^−/+^ mice exhibited increased scales and higher PASI scores in comparison to *Fbp1*^+/+^ mice (Supplementary Fig. 4g, h). Histological analysis demonstrated aggravated psoriasiform hyperplasia and increased epidermis thickness in *Fbp1*^−/+^ mice compared with *Fbp1*^+/+^ mice (Fig. 7j,k). Furthermore, the skin of *Fbp1*^−/+^ mice showed lower *Loricrin* expression and higher Ki-67 numbers than that of *Fbp1*^+/+^ mice (Fig. 7l, m), demonstrating decreased differentiation and increased proliferation in psoriasiform lesions of *Fbp1*^−/+^ mice. The number of infiltrated immune cells was also increased in psoriasis-like skin lesions of *Fbp1*^−/+^ mice (Fig. 7n and Supplementary Fig. 4i). These results suggest that *Fbp1* deficiency aggravates psoriasis-like phenotype in imiquimod-treated mice.

We next examined whether 2-DG or 2-HC alleviated psoriasis-like phenotype in *Fbp1*^−/+^ mice. Indeed, topical application of 2-DG or 2-HC decreased scale, inhibited psoriasiform hyperplasia and skin thickening (Supplementary Fig. 4g, h and Fig. 7j, k) compared to vehicle-treated *Fbp1*^−/+^ mice. The skin lesions of 2-DG or 2-HC treated *Fbp1*^−/+^ mice showed higher *Loricrin* expression and lower Ki-67 staining than that of vehicle-treated *Fbp1*^−/+^ mice (Fig. 7l, m). The infiltration of leukocytes (CD45^+^), macrophages (F4/80^+^) and T cells (CD4^+^) was also attenuated by 2-DG or 2-HC treatment compared to vehicle treatment (Fig. 7n and Supplementary Fig. 4i). Thus, *Fbp1* loss aggravates psoriasis-like skin lesions partially through glycolysis and acetyl-CoA production.

## Discussion

**Fig. 8:**
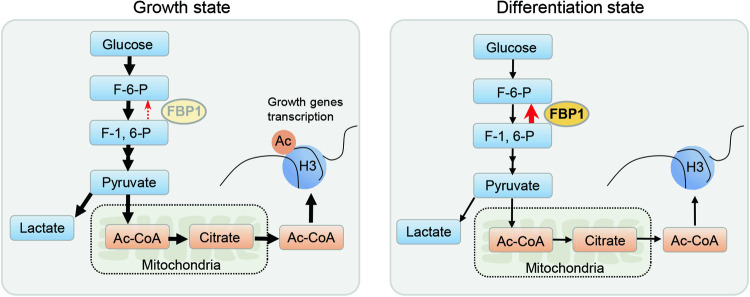
Working model for cell state transition controlled by FBP1. FBP1 controls keratinocyte state through regulating glycolysis and acetyl-CoA production, which determines histone acetylation level at growth genes.

The epidermis is a self-renewal tissue that protects the body against environmental insults and excess water loss. Emerging studies reveal glycolytic enzymes contribute to this essential process [34]. However, the role of gluconeogenic enzymes in epidermis has not been explored. Here we provide genetic evidence that the gluconeogenic enzyme FBP1 participates in epidermal homeostasis and response to UVB irradiation. *FBP1* loss promotes proliferation and maintains dedifferentiation state of keratinocytes. Mechanistically, *FBP1* deficiency facilitates glycolysis-mediated acetyl-CoA production, thereby resulting in increased H3K9 acetylation and enhanced transcription of proliferation genes (Fig. 8). Moreover, *Fbp1* loss aggravates psoriasis-like skin lesions through glycolysis and acetyl-CoA in the mice imiquimod psoriasis model.

We provide several evidences that FBP1 regulates proliferation and differentiation of keratinocytes. First, *FBP1* expression is induced by UVB irradiation, especially in upper epidermis and parallels the expression of epidermal differentiation marker. Second, genetic ablation of *Fbp1* in mice results in local epidermal hyperplasia featured by increased proliferation and decreased differentiation of keratinocytes. Third, *FBP1* loss promotes proliferation and maintains the dedifferentiation state of keratinocytes in vitro. Additionally, we also found *FBP1* depletion abolishes expression of inflammation cytokines *IL1B* and *IL-6* in keratinocytes. As IL-1 and IL-6 play key roles in inflammatory responses to triggers and wound healing [35–39], FBP1 may also participate in these processes which need further investigation.

Metabolites generated during glycolytic and oxidative processes are utilized in enzymatic reactions leading to epigenetic modifications and transcriptional regulation, thus facilitating the transition from one cell type to another (e.g., differentiation) [40–42]. Metabolic reprogramming of epigenetics is involved in a variety of processes such as cell fate, development, cancer and trained immunity [40, 43, 44]. The link between metabolism and epigenetic machinery in keratinocytes is less clarified. Here we find the increased metabolite acetyl-CoA accounts for the elevated H3K9 acetylation in *FBP1* deficient keratinocytes, which is essential for growth-related genes transcription and cell proliferation.

Psoriasis is a common skin disorder characterized by abnormal immune cell infiltration and cytokine-driven epidermal hyperplasia [45]. Although many therapies can reduce symptoms, psoriasis has no known cure [1]. Several studies have shown that dysregulated metabolic pathways are linked to psoriasis pathogenesis. Compared with healthy individuals, patients with psoriasis have higher levels of lactic acid and amino acids, indicating the involvement of glycolysis and amino acid metabolic pathway [46, 47]. Indeed, dimethyl fumarate, an immunomodulatory drug used to treat psoriasis and multiple sclerosis, was recently shown to mediate anti-inflammatory effects through inactivating the glycolytic enzyme glyceraldehyde 3-phosphate dehydrogenase (GAPDH) in activated myeloid and lymphoid cells [48]. Here we find *FBP1* is dramatically reduced in human psoriatic lesions and in skin of mice imiquimod psoriasis model, in vivo study demonstrates *Fbp1* deficiency facilitates psoriasis-like skin lesions development through glycolysis and acetyl-CoA. *Fbp1* deficiency also promotes the inflammatory infiltration in animal models of psoriasis in glycolysis and acetyl-CoA-dependent manner. Consistent with previously reported roles for glycolysis in T lymphocyte activation [49, 50], we speculate that *Fbp1* deficiency might also facilitate glycolysis in other cell types in the skin, including infiltrating lymphocytes, and thereby aggravate inflammation in vivo. Thus, targeting glycolysis or acetyl-CoA production may represent potential treatment for psoriasis patients with reduced *FBP1* expression in skin lesions. Additionally, the glycolytic enzyme *PFKFB3* is overexpressed in the skin of psoriasis patients, and in the skin of IMQ-mouse model [5, 51], which may well be involved in the activation of glycolysis, together with the downregulation of *FBP1*. Moreover, consistent with the decreased expression of *FBP1* in clear cell renal cell carcinoma, breast cancer and hepatocellular carcinoma [13–16], we find *FBP1* is also downregulated in SCC, implicating the tumor suppressive role of FBP1 in skin carcinoma. Furthermore, the exact role of FBP1 in other proliferative skin diseases also deserves future exploration.

Using pathway specific inhibitors, we find *FBP1* expression is partially regulated by p38 MAPK pathways and NF-κB pathways. However, other mechanisms may also exist as these inhibitors cannot completely silence *FBP1* expression. For example, promoter methylation may also participate in regulating FBP1 levels in skin as *FBP1* promoter exhibits higher methylation in hepatocellular carcinoma than in normal liver tissues [15]. Importantly, elucidating the reason why FBP1 is downregulated in psoriasis may help to restore FBP1 level and alleviate psoriasis in the future.

Since *Fbp1* gene is inactivated in the whole mouse in this study, some of the observed phenotype in *Fbp1*^−/+^ mice might be due to the deficiency of the enzyme in other types of cells and not only in keratinocytes, for example, immune cells, thus keratinocyte-specific ablation of *Fbp1* in mice should be conducted in the future to elucidate the exact role of FBP1 in keratinocytes in vivo.

In summary, our study reveals that FBP1 plays critical role in normal skin homeostasis and response to external stimuli, and identifies that *FBP1* loss promotes cell state transition and facilitates psoriasis-like skin lesions through metabolite-mediated epigenetic alteration.

## Material and methods

### Animals

*Fbp1* heterozygous (*Fbp1*^−/+^) mice, generated through the CRISPR-Cas9-mediated deletion of exon 2 to exon 4 of the *Fbp1* gene, were bought from GemPharmatech (Nanjing, Jiangsu, China). *Fbp1* homozygous (*Fbp1*^−/−^) mice were obtained by crossing female and male *Fbp1*^−/+^ mice. All mice were on the C57BL/6 J background and maintained under specific pathogen-free conditions. All experimental procedures in mice were approved by the Laboratory Animal Center of Chinese Academy of Military Medical Sciences and complied with all relevant ethical regulations. They were conducted according to the NIH Guide for the Care and Use of Laboratory Animals. For the imiquimod-induced psoriasiform hyperplasia model, 6-week-old mice were shaved and chemically depilated. The shaved dorsal-skin samples were treated topically with 50 mg of 5% imiquimod (Aldara, 3 M Pharmaceuticals) daily. Skin tissues were harvested after 5 or 8 days of treatment. 2-DG (D807272, Macklin, Shanghai, China) or 2-HC (P815515, Macklin, Shanghai, China) was dissolved in water. For 2-DG or 2-HC treatment, mice were randomly assigned to receive 2-DG (0.5%, 100 μl), 2-HC (1%, 100 μl), or water vehicle control (100 μl), respectively. The treatment was applied topically immediately before imiquimod treatment.

### Cell culture and transfection

Human primary keratinocytes were bought from Biocell Biotechnology (Guangdong, China), and grown in ready-to-use keratinocyte growth medium (PromoCell, C-20011) supplemented with Supplement Mix (PromoCell, C-20011) and 0.06 mmol/L CaCl_2_. HaCaT cell lines were obtained from the American Type Culture Collection and cultured in calcium free MEM (Sigma, M0518) supplemented with 0.06 mmol/L CaCl_2_ and 10% FBS in which calcium was removed by Chelex 100 (Macklin, I832447). For differentiation of keratinocytes, CaCl_2_ was added to the medium to 1.2 mmol/L. For *IL-6* or *TP63* knockdown, cells were transfected with indicated siRNA using Lipofectamine 3000 (Invitrogen) reagent according to the manufacturer’s protocol. Cells were collected and indicated proteins were analyzed by western blot 72 h after transfection. SiRNA target sequences were listed in Supplementary Table. For adezmapimod (MCE, HY-10256) or BAY 11-7082 (MCE, HY-13453) treatment, cells were collected and indicated proteins were analyzed by western blot 48 h after treatment.

### Human epidermis equivalents

Keratinocytes harvested and suspended in ice-cold medium (PromoCell, C-20011) containing 1.2 mM calcium were seeded on polycarbonate culture inserts (0.4 μm pores in diameter, Snapwell permeable supports, Costar, 3407). After 24 h of incubation at 37 °C in a humidified atmosphere containing 5% CO_2_, cells were exposed to the air–liquid interface, and 50 μg/ml vitamin C (Sigma, A92902) and 10 ng/ml keratinocyte growth factor (PeproTech, 100-19) were added to the medium in the lower compartment. The medium was renewed every day during the air–liquid interface culture.

### UVB irradiation

The TL 20W/12RS fluorescent lamp (Philips, Eindhoven, Holland) was used to irradiate the mice, human epidermis equivalents or keratinocytes. The power of the lamp was 431 μW/cm^2^ detected by a UV-AB meter (Tenmars, TM-213). The mice or human epidermis equivalents were placed 40 cm below the light source and irradiated. Vertical movement of mice was restrained by a wire screen.

### RNA-seq data analysis

Reference genome and gene model annotation files were downloaded from genome website browser Ensembl directly. Indexes of the reference genome were built using Bowtie v2.0.6 and paired-end clean reads were aligned to the reference genome using TopHat v2.0.9. Cufflinks v2.1.1 was used to count the fragment numbers mapped of each gene. And then FPKM of each gene was calculated based on the length of the gene and fragments count mapped to this gene. Differential expression analysis between three groups (Control, UVB_24 h and UVB_72 h) was performed using the DESeq2 R package (1.32.0). Genes with Q-value < 0.001 and log2FC > 1 found by DESeq2 were selected. Correlation was determined using the cor. test function in R with method = “Pearson”. Pathway analyses were performed using GSEA software (4.0.3) and Cluster Profiler (4.4.2) package in R (4.1.1).

### Histology

Paraffin-embedded skin specimens were sectioned, stained with hematoxylin and eosin (H&E) and visualized using a light microscope (Nikon, Eclipse 80i, Japan). Epidermal thickness was measured using ImageJ software.

### Immunohistochemistry

For immunohistochemical analysis, 4% paraformaldehyde-fixed sections were blocked with 5% bovine serum albumin and incubated with primary antibodies against FBP1 (Proteintech, 12842-1-AP), keratin 10 (Abcam, ab76318), loricrin (Abcam, ab198994), IL-1β (Abclonal, A16288), IL-8 (Proteintech, 27095-1-AP), Ki-67 (Cell Signaling Technology, 12202), CD45 (Abcam, ab10558), F4/80 (Abcam, ab6640), CD4 (Abcam, ab183685) overnight at 4 °C. Tissue sections were incubated with biotinylated anti-rabbit IgG (Vector Laboratories, BA-1000) for 30 min at room temperature. The antigen–antibody binding was detected by 3,3′-diaminobenzidine (Solarbio, DA1016) system. Tissue sections were briefly immersed in hematoxylin for counterstaining and were covered with cover glasses.

### Immunocytochemistry

For immunocytochemical analysis, cells were fixed with 4% paraformaldehyde, permeabilized with 0.2% Triton-X-100/PBS and blocked with 5% bovine serum albumin. Cells were incubated with primary antibodies against H3K9-ac (Zen-bio, 340016) overnight at 4 °C. Cells were then incubated with the corresponding CoraLite488-conjugated goat anti-rabbit IgG (Proteintech, SA00013) for 1 h at room temperature. Cells were mounted with mounting medium with DAPI. Images were taken using Cytation 5 Cell Imaging Multimode Reader (Biotek).

### Western blots

For western blots, samples of cells or tissues were separated on SDS–PAGE gels, transferred to PVDF membranes and probed with primary antibodies to the following proteins: keratin 10 (Abcam, ab76318), involucrin (Santa Cruz Biotechnology, sc-21748), FBP1 (Proteintech, 12842-1-AP), β-actin (Proteintech, 20536-1-AP), IL-1β (Proteintech, 16806-1-AP), IL-6 (Proteintech, 21865-1-AP), H3K9-ac (Zen-bio, 340016), β-tubulin (Zen-bio, 200608), H3K27-ac (Abclonal, A7253), p63 (Zen-bio, 381215). Samples were then stained with secondary antibodies conjugated to HRP at a dilution of 1:5000 (Santa Cruz, donkey anti-rabbit, SC2077 and donkey anti-mouse, SC2096) and detected with an ECL system (PerkinElmer, NEL104001EA). Full and uncropped western blots can be found in the Supplementary Material.

### Reverse transcription quantitative real-time PCR (RT-qPCR)

Total RNA was extracted from epidermal preparations using TRIzol Reagent (Invitrogen, 15596026). Two micrograms total RNA was reverse transcribed using TransScript One-Step gDNA Removal and cDNA Synthesis SuperMix (TransGen Biotech, AT311-02). Gene expression analysis was performed on Applied Biosystems Real-Time PCR System (Thermo Fisher). Gene expression was analyzed using TransStart Green qPCR SuperMix (TransGen Biotech, AQ101-01) at the following conditions: 95 °C for 2 min, followed by 40 cycles of denaturation (95 °C for 5 s), annealing and extension (30 s at temperature experimentally determined for each primer pair). Amplification differences between samples and controls were calculated based on the Ct (ΔΔCt) method and normalized to *GAPDH*. RT-qPCR primers were listed in Supplementary Table.

### Lentivirus packaging and infection

Lentiviral vectors (GV248) carrying *FBP1* shRNA were from GeneChem Company (China). The vector was transfected into HEK293T cells together with pSPAX.2 and pMD.2G for 24 h, and cell culture media were collected and filtered. The viral particles were precipitated by Polyethylene glycol 8000 (0.05 g/ml) and then used to infect cells. Cells were cultured in medium containing 2 μg/ml puromycin (MCE, HY-B1743A) for the selection of stable clones 3 days post infection. The positive clones were identified and verified by western blots. The *FBP1* shRNA target sequences were as follows: shFBP1-1: 5′-CCTTGATGGATCTTCCAACAT-3′, shFBP1-2: 5′-CGACCTGGTTATGAACATGTT-3′, shFBP1-3: 5′-CAGCAGTCAAAGCCATCTCTT-3′.

### Glucose uptake assay

Glucose uptake in HaCaT cells was measured by analysis of 2-NBDG taken up by cells using 2-NBDG Glucose Uptake Assay Kit (BioVision, K682-50). Images were taken using Cytation 5 Cell Imaging Multimode Reader (Biotek) with an excitation wavelength of 485 nm and an emission wavelength of 535 nm.

### Lactate measurement

HaCaT cells were incubated for an additional 24 h in fresh growth medium 3 days after plating. Medium was collected on ice, centrifuged for 10 min at 2500 r.p.m. at 4 °C and lactate contents were measured by cobas c311 analyzer (Roche) according to the operator’s manual. The values were normalized to the total cellular protein level.

### ECAR measurement

ECAR was measured using Agilent Seahorse XF Glycolysis Stress Test Kit (Agilent Technologies, 103020-100). Briefly, HaCaT cells were resuspended with MEM medium and plated into Seahorse XFe Microplate by 10,000 cells/well, then the plate was incubated at 37 °C overnight. Glucose (10 mM, Sigma, G8644), oligomycin (10 µM, Sigma, 75351) and 2-DG (50 mM, Sigma, D8375) solution was sequentially added into the appropriate ports of a hydrated sensor cartridge. To evaluate ECAR, the calibration plate was replaced with the cell culture microplate, and the “Start” button was pressed.

### Acetyl-CoA assay

Acetyl-CoA levels in HaCaT cells were determined by Acetyl-Coenzyme A Assay Kit (Sigma, MAK039). Fluorescence intensity was detected using Cytation 5 Cell Imaging Multimode Reader (Biotek) with an excitation wavelength of 535 nm and an emission wavelength of 587 nm. Values were expressed as fold differences compared to control.

### Colony formation assay

For colony formation assay, HaCaT cells were diluted to the single cell suspension and 1000 cells were cultured in every well of six-well plate at 5% CO_2_ incubator for 2 weeks. Then the colonies were stained with 0.1% crystal violet and counted.

### Chromatin immunoprecipitation assay and qPCR

Chromatin immunoprecipitation assays were performed using the Simple ChIP Plus Enzymatic Chromatin IP Kit (Cell Signaling Technology, 9005) according to the product manual. Quantitative Real-Time PCR was used to measure the amount of enrichment of a particular DNA sequence by histone H3 (Cell Signaling Technology, 4620) or H3K9ac (Zen-bio, 340016) immunoprecipitation. Ct value detected from H3K9ac immunoprecipitates were normalized by Ct value from histone H3 immunoprecipitates. Amplification differences between samples and controls were calculated based on the Ct (ΔΔCt) method. QPCR primers were list in Supplementary Table.

### Statistical analysis

The statistical significance of differences between two groups was calculated with the two-tailed Student’s *t-*test, and error bars represent standard deviation of the mean (s.d.). Statistical analyses were performed using GraphPad Prism 7. Data are shown as mean ± s.d. *P* < 0.05 was considered statistically significant.

### Supplementary information

Supplementary Information

Original Western Blots

## Data Availability

Gene expression profile of human epidermal equivalents has been deposited into Figshare (10.6084/m9.figshare.21299496.v1).
