# Supplementary material for: FBP1 orchestrates keratinocyte proliferation/differentiation and suppresses psoriasis through metabolic control of histone acetylation
Source: Cell Death Dis. 2024 Jun 4;15(6):392. doi: 10.1038/s41419-024-06706-6 (PMC11150480; doi:10.1038/s41419-024-06706-6)
Supplement: Supplementary file 1 — Supplementary Information [file 41419_2024_6706_MOESM1_ESM.pdf]

SUPPLEMENTARY INFORMATION

FBP1 orchestrates keratinocyte proliferation/differentiation and suppresses psoriasis through metabolic control of histone acetylation

Pengfei Zhang[1&], Ju Yang[2&], Xiong Liu[3&], Congshu Huang[1], Yuandong Tao[4], Pan Shen[1], Zhijie Bai[1], Chengrong Xiao[1], Lei Zhou[1], Gaofu Li[1], Li Zhang[3], Wei Zhou[1*], Yue Gao[1*]

SUPPLEMENTARY FIGURES

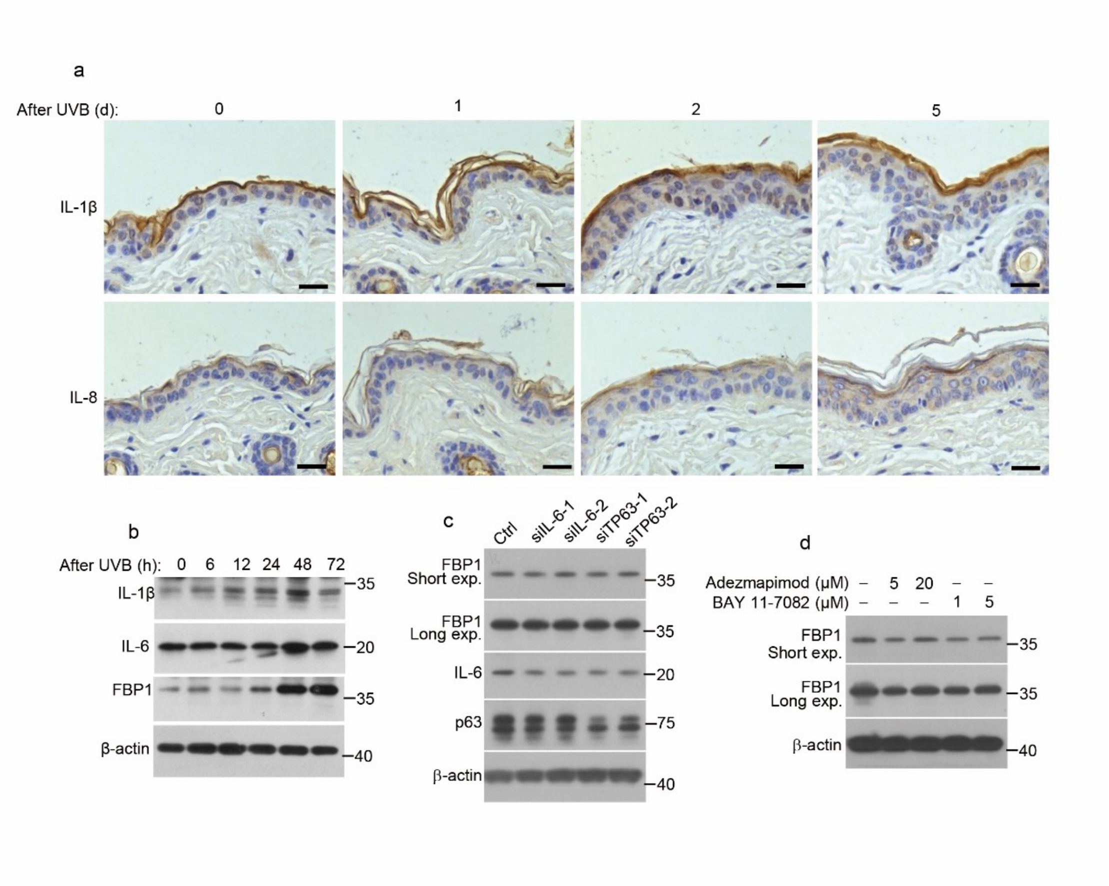

Supplementary Fig. 1 a, Representative immunohistochemical staining for IL-1β and IL-8 of mice skin collected at indicated days after irradiation. Scale bars: 25 μm. **b**, Western blot analysis of IL-1β, IL-6, FBP1 and β-actin in HaCaT cells at indicated time after UVB irradiation. **c**, Western blot analysis of the expression of indicated proteins in keratinocytes 72 hours after transfection of *IL-6* siRNA (siIL-6-1, siIL-6-2) or *TP63* siRNA (siTP63-1, siTP63-2). **d**, Western blot analysis of the expression of

indicated proteins in keratinocytes 48 hours after adezmapimod or BAY 11-7082 treatment.

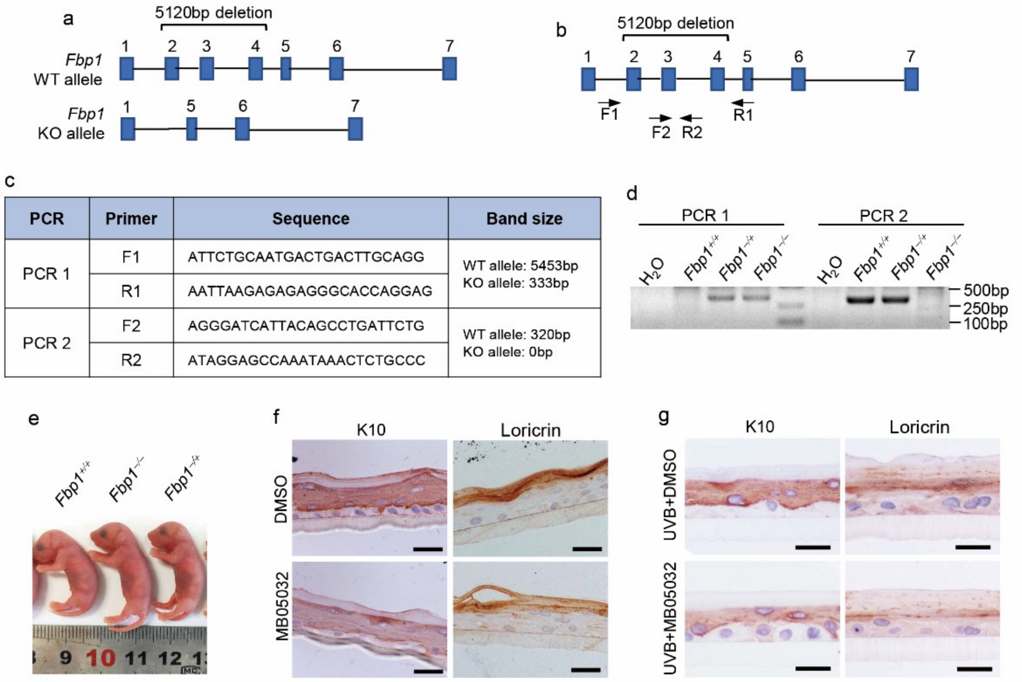

Supplementary Fig. 2 **a**, Schematic diagram for *Fbp1* deletion in mice. **b**, Schematic diagram for genotyping primers of *Fbp1*. **c**, Sequence of genotyping primers of *Fbp1*. **d**, PCR detection of a deletion in the *Fbp1* gene. Genomic DNA from *Fbp1*$^{+/+}$, *Fbp1*$^{-/+}$ and *Fbp1*$^{-/-}$ mice were amplified with primers shown in c and detected by agarose gel electrophoresis. **e**, Photos of neonatal *Fbp1*$^{+/+}$, *Fbp1*$^{-/+}$ and *Fbp1*$^{-/-}$ littermates. **f**, Representative immunohistochemical staining (K10, loricrin) of DMSO or MB05032 (40 μM) treated human epidermal equivalents. Scale bars: 25 μm. **g**, Human epidermal equivalents were exposed to 500 mJ/cm^2 UVB irradiation and then treated with DMSO or MB05032 (40 μM) for 3 days and collected. Representative immunohistochemical staining for K10 and loricrin were shown. Scale bars: 25 μm.

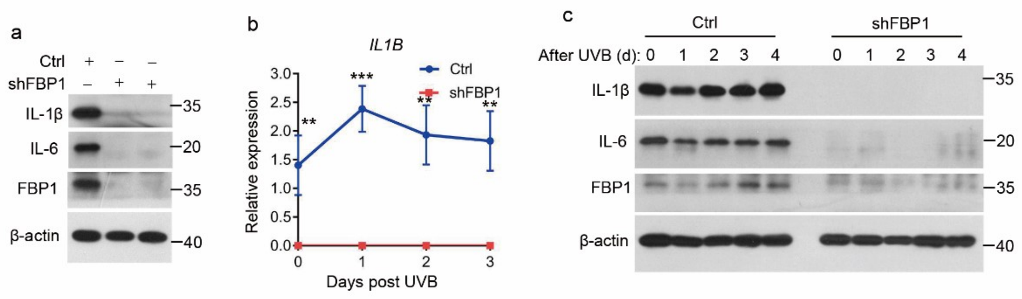

Supplementary Fig. 3 a, Western blot analysis of the IL-1β, IL-6, FBP1 and β-actin in Ctrl and shFBP1 HaCaT cells. **b**, Relative mRNA levels of *IL-1β* in Ctrl and shFBP1 HaCaT cells at indicated days after UVB irradiation (50 mJ/cm^2). n = 3 independent experiments. **c**, Western blot analysis of the IL-1β, IL-6, FBP1 and β-actin in Ctrl and shFBP1 HaCaT cells at indicated days after UVB irradiation (50 mJ/cm^2). Data are shown as mean ± s.d. Statistical analyses in (**b**) were performed with Student's t-tests. ** $p< 0.01$, *** $p< 0.001$.

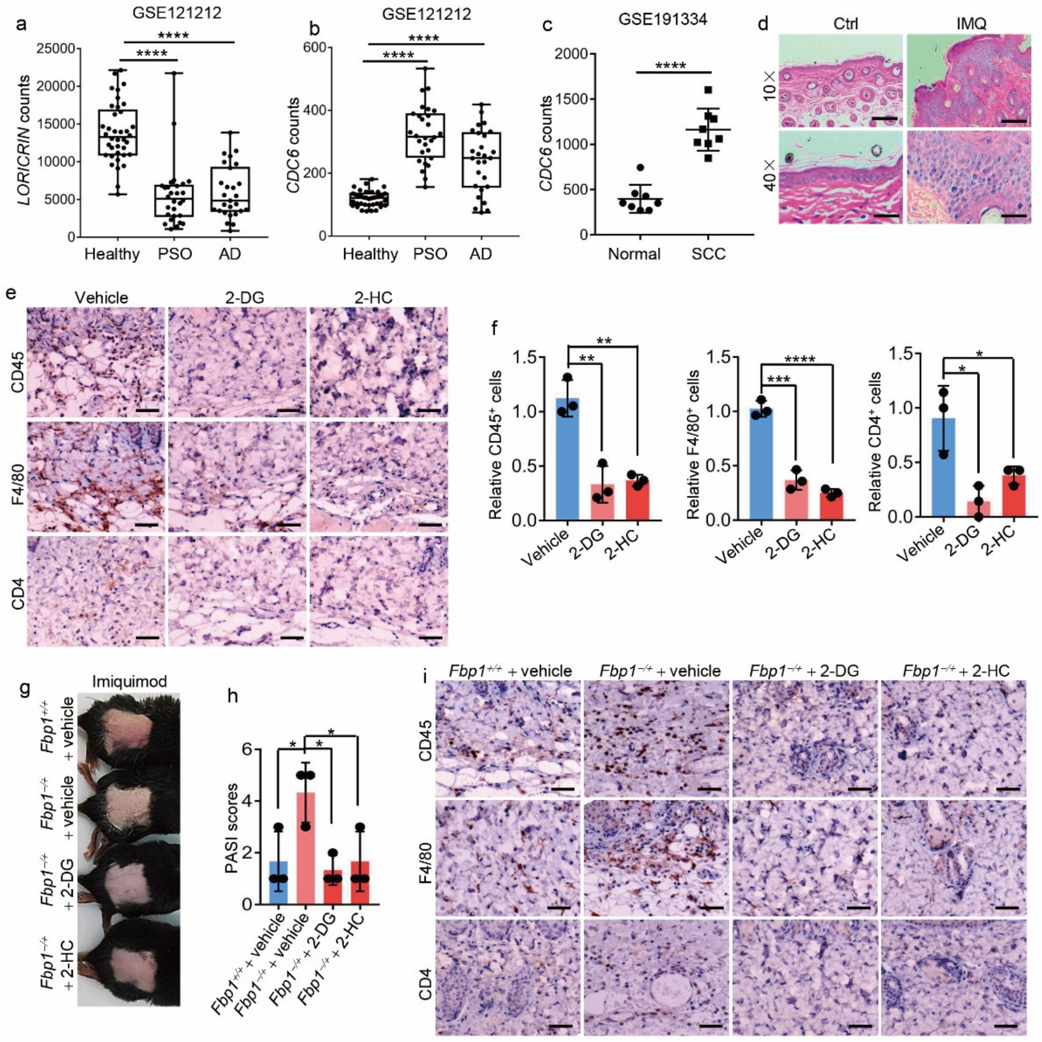

Supplementary Fig. 4 a, **b**, *LORICRIN* (**a**) or *CDC6* (**b**) levels in healthy skin, psoriatic lesions or atopic dermatitis lesions based on RNA-Seq datasets from public databases GSE121212. **c**, *CDC6* levels in normal skin or squamous cell carcinoma based on GSE191334. **d**, Representative H&E staining of skin from Ctrl or imiquimod treated mice. Scale bars in upper images: 200 μm, scale bars in lower images: 50 μm. **e**, Immunohistochemical staining for CD45, F4/80 or CD4 of imiquimod induced skin lesions from vehicle, 2-DG or 2-HC pretreated mice. Scale bars: 50 μm. **f**, Relative leukocytes ($CD45^+$), macrophages ($F4/80^+$) or T cells ($CD4^+$) numbers of imiquimod induced skin lesions (8 days) from vehicle, 2-DG or 2-HC pretreated mice. n = 3 mice per group. **g**, Photos of imiquimod induced skin lesions (5 days) from vehicle pretreated *Fbp1*$^{+/+}$ mice, vehicle, 2-DG or 2-HC pretreated

Fbp1$^{-/+}$ mice. **h**, The Psoriasis Area and Severity Index (PASI) score for imiquimod induced skin lesions (5 days) from vehicle pretreated *Fbp1*$^{+/+}$ mice, vehicle, 2-DG or 2-HC pretreated *Fbp1*$^{-/+}$ mice. n = 3 mice per group. **i**, Immunohistochemical staining for CD45, F4/80 or CD4 of imiquimod induced skin lesions (8 days) from vehicle pretreated *Fbp1*$^{+/+}$ mice, vehicle, 2-DG or 2-HC pretreated *Fbp1*$^{-/+}$ mice. Scale bars: 50 μm. Data are shown as mean ± s.d. Statistical analyses in (**a**), (**b**), (**c**), (**f**) and (**h**) were performed with Student's t-tests. * p< 0.05, ** p< 0.01, *** p< 0.001, **** p< 0.0001.

SUPPLEMENTARY TABLES

Supplementary table 1. RT-qPCR primers used in this study.

Gene	Forward primers	Reverse Primers
CYP24A1	GCAGCCTAGTGCAGATTT	ATTCACCCAGAACTGTTG
FBP1	ACCCTGCCGTCACTGAGTA	GCCCCATAAGGAGCTGAAT
HRNR	TCGAGACCATAACAAGAAAGTGG	GTGAGTGTCATCTCTCAGCTTTG
GAST	ATGCAGCGACTATGTGTGTATG	GCCCCTGTACCTAAGGGTG
TNFRSF10C	TGCACAGAGGGTGTGGATTAC	ATTCCGGAAGGTGCCTTCTTT
CDC6	CCAGGCACAGGCTACAATCAG	AACAGGTTACGGTTTGGACATT
TP63	GGACCAGCAGATTCAGAACGG	AGGACACGTCGAAACTGTGC
MYC	GGCTCCTGGCAAAAGGTCA	CTGCGTAGTTGTGCTGATGT
IL1B	CTGTCCTGCGTGTTGAAAGA	TTCTGCTTGAGAGGTGCTGA

Supplementary table 2. The target sequences of lentiviral shRNAs used in this study.

siRNA	Target sequence
siIL-6-1	GCAAAGAGGCACTGGCAGA
siIL-6-2	AGACTTGCCTGGTGAAAAT
siTP63-1	GGACAGCAGCATTGATCAA
siTP63-2	CCACTGAACTGAAGAAACT
