# Supplementary figures and images for: FBP1 orchestrates keratinocyte proliferation/differentiation and suppresses psoriasis through metabolic control of histone acetylation
Source: Cell Death Dis. 2024 Jun 4;15(6):392. doi: 10.1038/s41419-024-06706-6 (PMC11150480; doi:10.1038/s41419-024-06706-6)

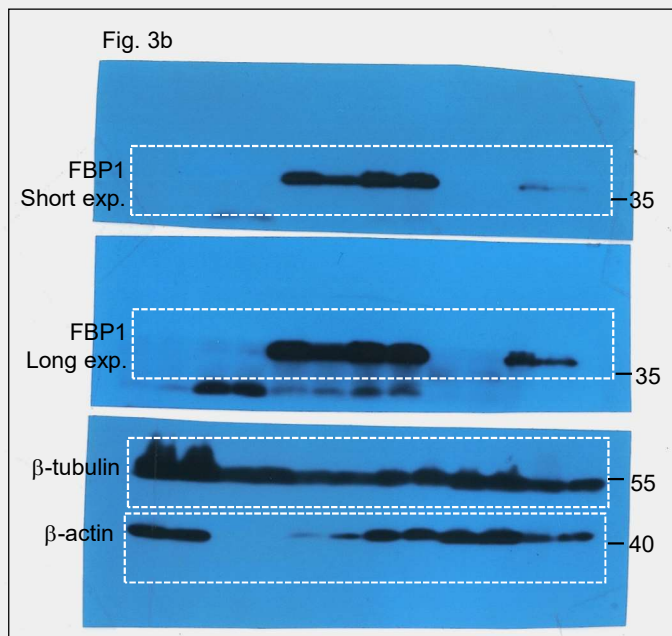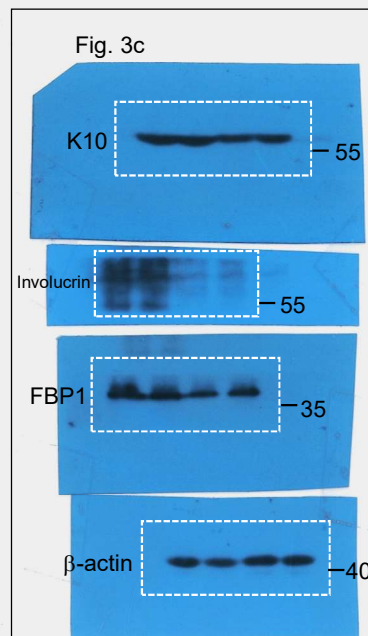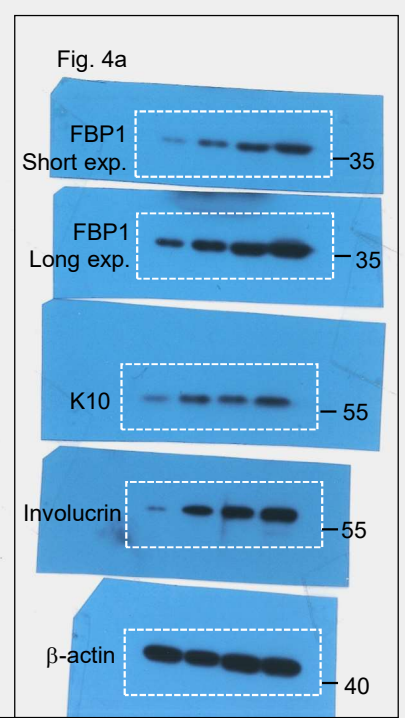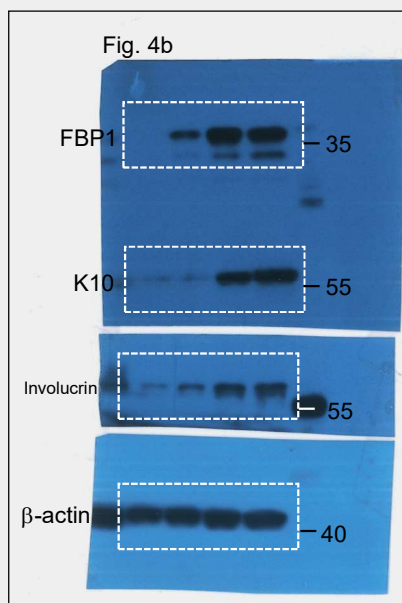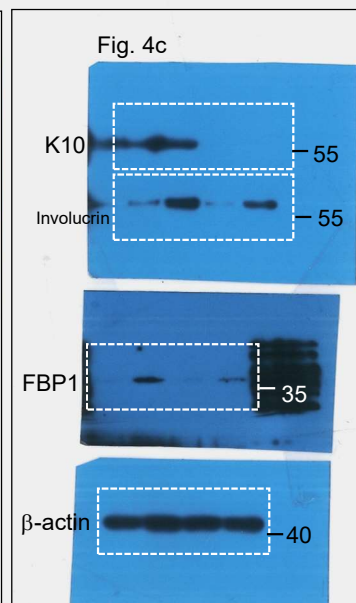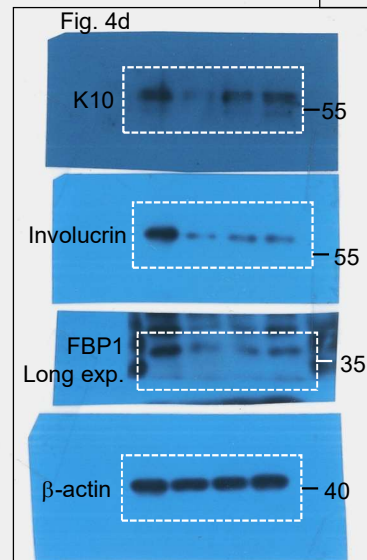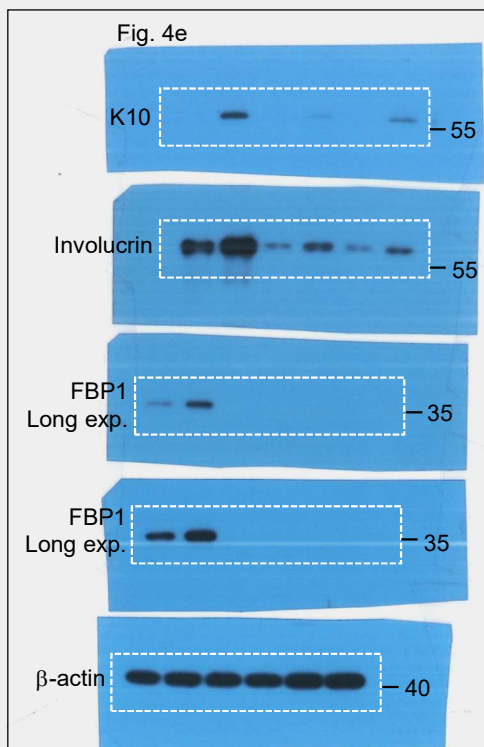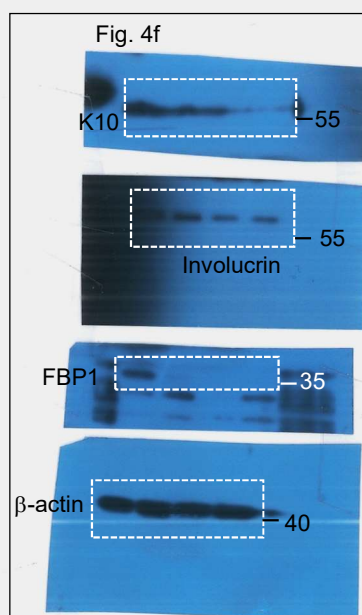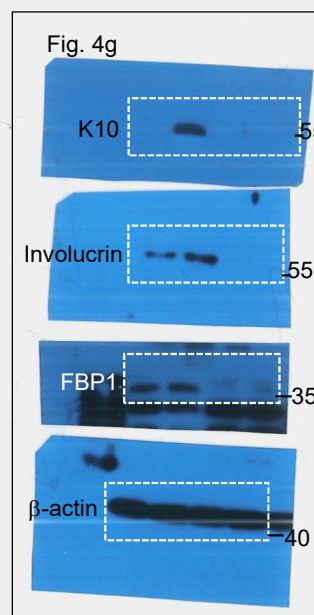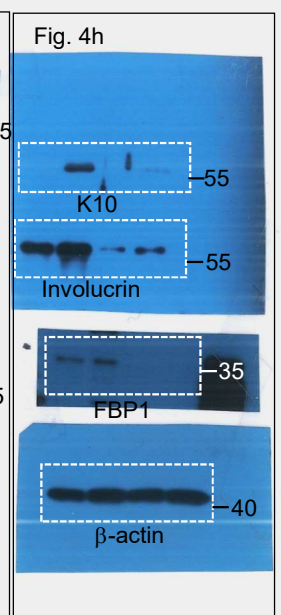

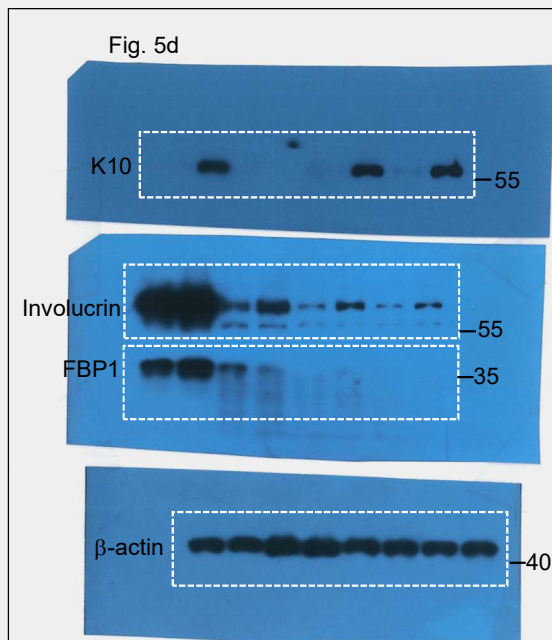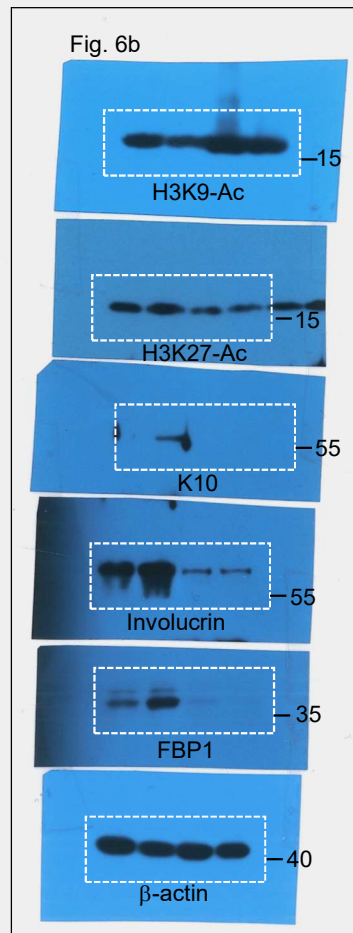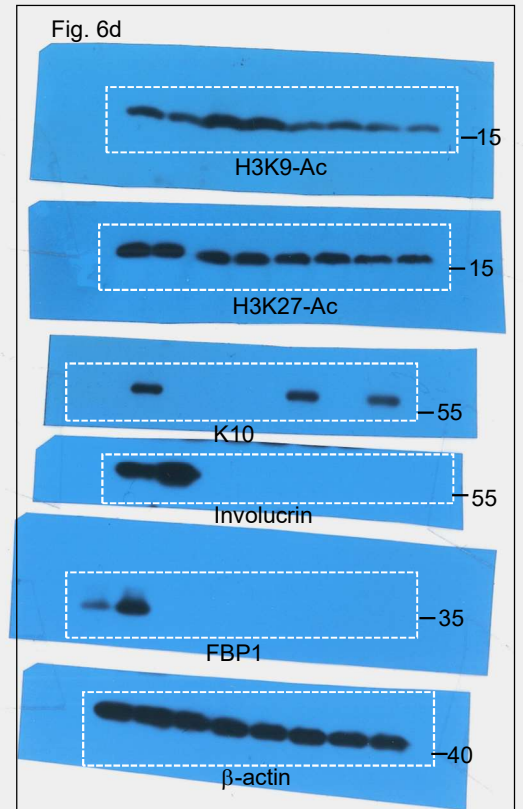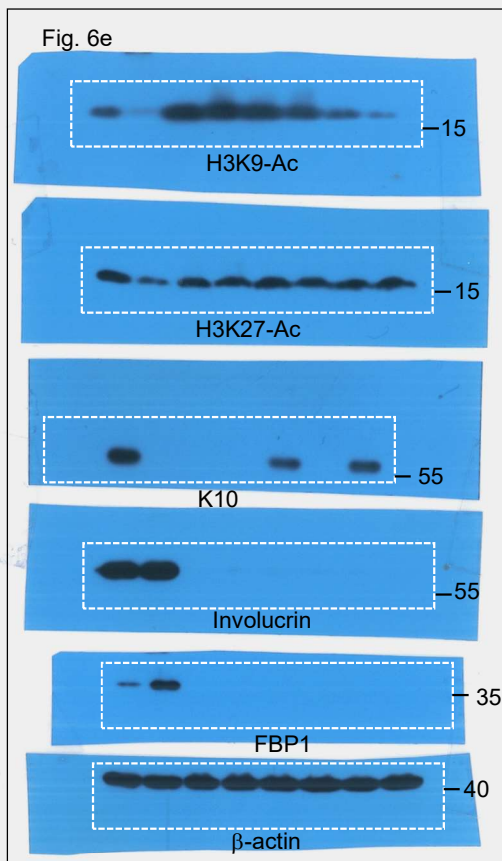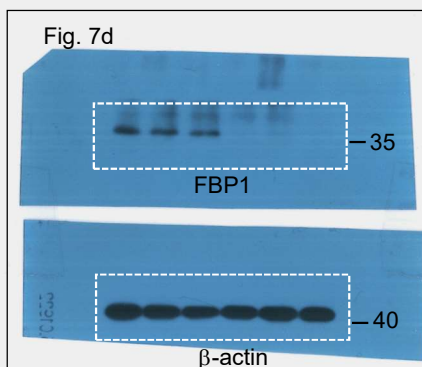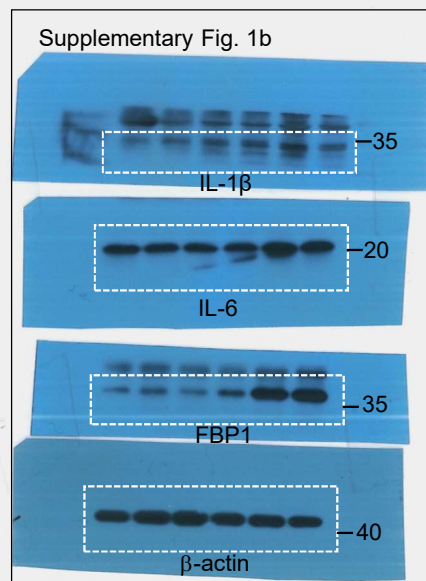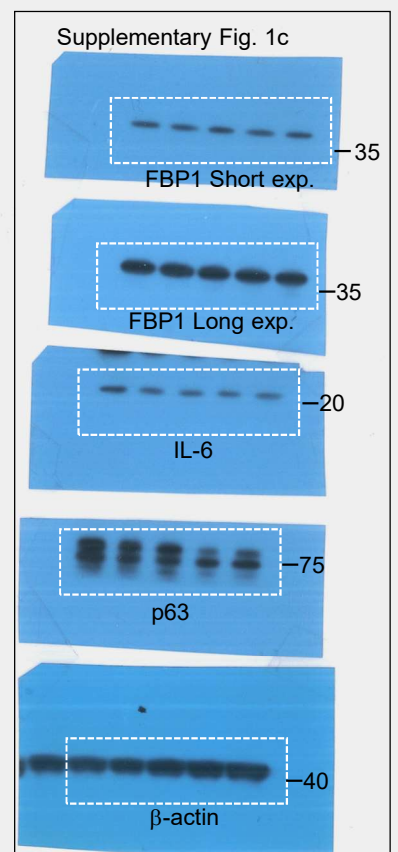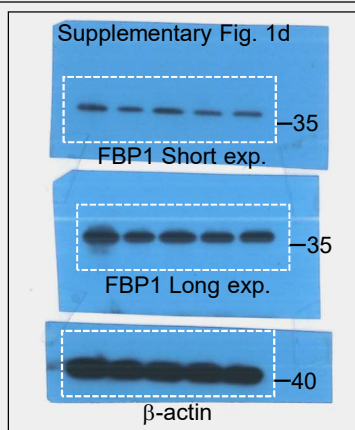

Supplementary Fig. 3a

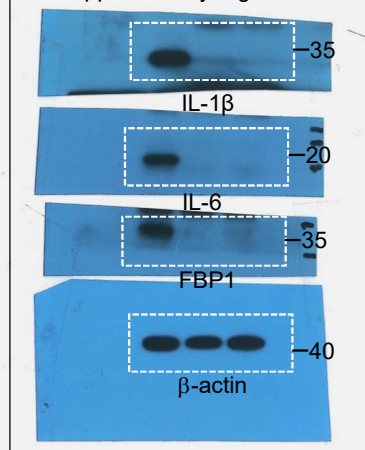

Supplementary Fig. 3c

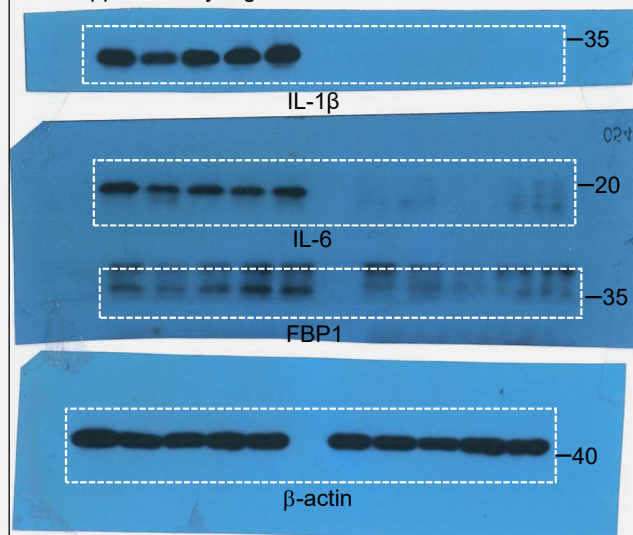

Supplement: Supplementary file 2 — Original Western Blots [file 41419_2024_6706_MOESM2_ESM.pdf]
